# Coordinated Multi-Scenario Optimization Strategy for Park Photovoltaic Storage Based on Master–Slave Game

**DOI:** 10.3390/s24155042

**Published:** 2024-08-04

**Authors:** Jiang Wang, Jinchen Lan, Lianhui Wang, Yan Lin, Meimei Hao, Yan Zhang, Yang Xiang, Liang Qin

**Affiliations:** 1 Hubei Key Laboratory of Power Equipment & System Security for Integrated Energy, Wuhan 430072, China; wangjangi@whu.edu.cn (J.W.); yangxiang@whu.edu.cn (Y.X.); 2 School of Electrical Engineering and Automation, Wuhan University, Wuhan 430072, China; 3 State Grid Fujian Electric Power Co., Ltd., Electric Power Science Research Institute, Fuzhou 350007, China; 18272029096@163.com (J.L.); lyepri@163.com (Y.L.); 4 State Grid Fujian Electric Power Co., Ltd., Fuzhou 350007, China; 13655593886@163.com (L.W.); 15231289390@163.com (M.H.); 18299693913@163.com (Y.Z.)

**Keywords:** photovoltaic storage park, multi-application scenarios, master–slave game, dual-level optimization, economic analysis

## Abstract

Optimizing the operation of photovoltaic (PV) storage systems is crucial for meeting the load demands of parks while minimizing curtailment and enhancing economic efficiency. This paper proposes a multi-scenario collaborative optimization strategy for PV storage systems based on a master–slave game model. Three types of energy storage system (ESS) application scenarios are designed to comprehensively stabilize PV fluctuations, compensate for load transfers, and participate in the frequency regulation (FR) market, thereby optimizing the overall operational strategy of PV storage systems in parks. The upper-level objective is to maximize the park operators’ profit, while the lower-level objective is to minimize the user’s power supply costs. Case studies demonstrate that this strategy can significantly increase the economic benefits for park operators by 25.8%, reduce user electricity expenditures by 5.27%, and lower curtailment through a load response mechanism, thereby promoting the development and construction of PV storage parks.

## 1. Introduction

In the context of global green and low-carbon transformation, the development of park-type PV systems has become a crucial measure to increase the proportion of distributed power sources in suburban areas [1]. The large-scale integration of PVs has intensified temporal randomness, spatial dispersion, and high-power disturbances within the power system. Current policies mandate that park PVs be equipped with ESSs to mitigate high-power fluctuations. However, ESSs often fail to fully utilize their regulatory potential when applied in a single scenario [2], resulting in underutilization and extended investment return cycles, thereby impeding the development of the PV storage industry. When ESSs simultaneously handle multiple application scenarios, the coupling of various applications increases scheduling complexity [3]. Therefore, it is imperative to explore collaborative optimization strategies for multiple application scenarios involving PV storage systems. These strategies aim to optimize the economic benefits for park operators and reduce power supply costs for users. This study will examine three specific scenarios: smoothing photovoltaic fluctuations, compensating for load transfer, and participating in the frequency regulation market. It will comprehensively address the interdependencies among these scenarios to ensure the efficient utilization of ESSs, ultimately achieving optimal operation of park PV storage systems.

Further investigation into the operational control of integrated ESSs across various application scenarios is essential. In response to the increasing peak–valley differences in commercial parks, Ref. [4] proposes a variable-parameter power difference control strategy for peak shaving and valley filling, establishing control strategies for ESSs based on different load intervals and state-of-charge (SOC) intervals. Ref. [5] presents a demand-side response model that combines combinatorial programming with particle swarm optimization, aiming to evaluate its impact on the economic benefits and ESS configuration of microgrids. Ref. [6] develops an equivalent value evaluation model for ESS applications, focusing on peak shaving, valley filling, and the smoothing of new energy fluctuations. This includes two equivalent evaluation models based on power difference and prediction accuracy. Refs. [7,8] suggests a revenue optimization method for ESSs participating in energy management within PV storage systems. Refs. [9,10] employ wavelet analysis to suppress photovoltaic power fluctuations and enhance photovoltaic output stability. In summary, ESSs offer diverse application modes that significantly improve the grid’s capacity to absorb new energy across multiple scenarios. Nevertheless, further research is needed on the coordinated optimization and energy distribution of PV storage systems across various application scenarios.

In multi-application scenarios involving optical storage in parks, it is crucial to balance the interests of both operators and users. Refs. [11,12] explore an optimization model for electricity pricing based on demand response, leveraging price control with multiple stakeholders to enhance the economic benefits for park operators. Refs. [13,14] focus on commercial parks, optimizing ESSs in the medium to long term by coordinating upper and lower levels to maximize energy utilization efficiency, considering seasonal differences in user load. Refs. [15,16] examine the energy sharing and trading mechanisms among multiple microgrids, employing cooperative game coordination to reduce energy consumption costs for demand-side multi-agents and improve economic benefits for park operators. The aforementioned literature has examined aspects such as demand response, electricity price optimization, and shared ESSs, which have enhanced the economic benefits and energy utilization efficiency of parks. However, they have not comprehensively considered the multiple application scenarios of park ESSs, and they lack a dual-focus approach that ensures mutually beneficial outcomes for both operators and users.

This paper comprehensively considers the output characteristics of photovoltaic storage parks and the transfer characteristics of loads. It integrates ESS participation in smoothing photovoltaic fluctuations, energy time shifting, and the frequency regulation market, proposing the concept of load transfer compensation. A master–slave game model for the joint operation of PVs and ESSs under multiple scenarios is established to optimize the coordination and efficiency of PV storage resource integration. The main contributions of this paper are as follows:

- Propose a multi-scenario collaborative optimization strategy for the operation of PV storage in parks, including smoothing photovoltaic fluctuations, load transfer compensation, and participation in the frequency regulation market, comprehensively summarizing the economic benefit model for the park.
- Establish a master–slave game model for the joint operation of PVs and ESSs in parks, with the upper-level goal of maximizing operator benefits and the lower-level goal of minimizing user power supply costs, achieving the optimization solution of the dual-level model.
- Present case studies validating the proposed optimization strategy, demonstrating a 25.8% increase in the economic benefits for park operators, a 5.27% reduction in user electricity expenses, and a decrease in the photovoltaic power curtailment rate to 1.478%. These results significantly enhance the overall economic efficiency of the park and the utilization efficiency of photovoltaic energy.

## 2. Multi-Application Scenarios for PV Storage Parks

**Figure 1:**
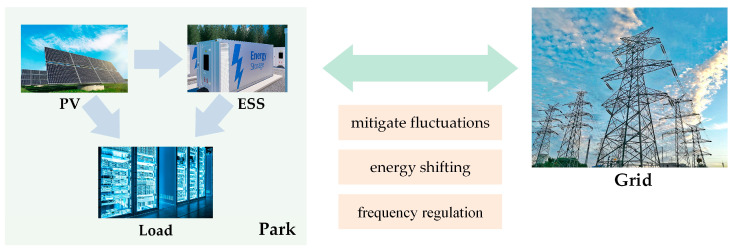
Combined operation mode of PV and ESS in park.

In the “self-generation and balance grid” mode, PV storage parks reduce the deviation between the actual and predicted photovoltaic output through ESSs. This mode enhances photovoltaic consumption rates and economic benefits by leveraging the energy time-shifting capabilities of storage or through electricity transactions with the main grid [17]. The ESS plays a crucial role in smoothing fluctuations, shifting energy over time, and participating in frequency regulation, as shown in Figure 1. This study investigates the coordinated optimization strategy for multi-scenario applications of ESSs, ensuring the safe and reliable operation of photovoltaic storage parks. It also aims to enhance economic benefits and increase the photovoltaic energy consumption rate.

### 2.1. Energy Storage Smoothing of PV Fluctuations

The characteristics of uncertainty, variability, and randomness associated with large-scale photovoltaic integration can adversely affect the power quality and stability of the grid. Moreover, the poor predictability of photovoltaic output power often results in prediction errors. Therefore, the rational design and optimization of ESSs can effectively mitigate prediction errors and address photovoltaic power fluctuations [18].

To ensure the stability of grid-connected power in PV storage parks, significant deviations from predicted values incur revenue penalties. However, leveraging the rapid response capabilities of ESSs to mitigate fluctuations in photovoltaic output can substantially minimize deviations and prevent penalties. This approach ultimately enhances the overall revenue of the park. Specifically, the benefits include the following:

(1) $$P_{i,t}^{\mathrm{pv}}-P_{i}^{\mathrm{pv}}+P_{i,t}^{e\_\mathrm{pv}}\leq \alpha P_{i}^{\mathrm{pv}}$$

where $P_{i,t}^{\mathrm{pv}}$ represents the actual output value of photovoltaic power during time period *t* within operation cycle *i*, $P_{i}^{\mathrm{pv}}$ denotes the predicted output value of photovoltaic power within operation cycle *i*, and $P_{i,t}^{e\_\mathrm{pv}}$ indicates the power signal for the ESS to smooth the photovoltaic fluctuation response. *α* defines the maximum allowable deviation range between the actual output and the predicted value. When the actual deviation exceeds this specified range, a penalty mechanism is activated, reducing the benefits. Within this allowable deviation range, no penalty is triggered.

Given the fluctuation characteristics of photovoltaic output, the design of the ESS prioritizes the suppression of these fluctuations to ensure power stability [19]. Consequently, the remaining capacity of the ESS available for other application scenarios within the cycle is calculated as follows:

(2) $$P_{i,t}^{\mathrm{ess}\_\mathrm{re}}=P_{i,t}^{\mathrm{ess}\_\max}-\max\{P_{i,t}^{e\_\mathrm{pv}}\}$$

where $P_{i,t}^{\mathrm{ess}\_\mathrm{re}}$ represents the remaining margin after the ESS meets the requirements for smoothing the photovoltaic fluctuations during cycle *i*, $P_{i,t}^{\mathrm{ess}\_\max}$ is the maximum value of the ESS output, and $\max\{P_{i,t}^{e\_\mathrm{pv}}\}$ represents the maximum power demand for the ESS to mitigate power fluctuations during period *t*. To ensure that the ESS has sufficient power to smooth power fluctuations, it is estimated based on the maximum possible ESS power in cycle *i*.

### 2.2. Energy Storage Participating in Energy Shifting

ESSs enhance power grid stability and economic benefits through electricity price arbitrage and peak shaving via energy transfer across different time periods. This study employs a variable power difference approach to manage ESS responses across diverse time intervals. Specifically, during load balancing operations, the ESS output mode is determined based on the deviation between the load demand and photovoltaic output limits [20].

When the photovoltaic output falls short of meeting the load demand, the ESS discharges or purchases electricity from the grid to bridge the deficit. Conversely, during surplus photovoltaic output, the system charges or sells electricity to optimize photovoltaic consumption. To assess the operational state of the ESS in energy time-shifting applications, it is essential to incorporate criteria for determining the charging and discharging status of the ESS as follows:

(3) $$\{\begin{array}{l} u_{t}^{\mathrm{bc}}=1,u_{t}^{\mathrm{bd}}=0,P_{t}^{\mathrm{load}}-P_{t}^{\mathrm{pv}\_\max}\leq 0\\ u_{t}^{\mathrm{bc}}=0,u_{t}^{\mathrm{bd}}=1,P_{t}^{\mathrm{load}}-P_{t}^{\mathrm{pv}\_\max}>0 \end{array}$$

where $u_{t}^{\mathrm{bc}}$ and $u_{t}^{\mathrm{bd}}$ are the charging and discharging indicators of the ESS participating in the energy market, $P_{t}^{\mathrm{load}}$ represents the load value during time period *t*, and $P_{t}^{\mathrm{pv}\_\max}$ denotes the photovoltaic output limit.

Based on the charge and discharge power limits of the ESS, its power capacity for participating in energy shifting is expressed as follows:

(4) $$\{\begin{array}{c} 0\leq P_{t}^{\mathrm{bc}}\leq u_{t}^{\mathrm{bc}}P^{\mathrm{ess}\_\max}\\ 0\leq P_{t}^{\mathrm{bd}}\leq u_{t}^{\mathrm{bd}}P^{\mathrm{ess}\_\max} \end{array}$$

where $P_{t}^{\mathrm{bc}}$ and $P_{t}^{\mathrm{bd}}$ represent the charging and discharging powers of the ESS involved in energy management.

The PV storage park utilizes the ESS to achieve a power balance between photovoltaic generation and load, integrating load response strategies to enhance photovoltaic consumption capacity and economic benefits. Therefore, this article introduces a load compensation mechanism alongside energy time shifting using the ESS, thereby augmenting its capability in peak shaving and valley filling within the park [21]. Thus, the load transfer amount can be calculated as follows:

(5) $$\Delta P_{t}^{\mathrm{load}}=P_{t}^{\mathrm{load}}-P_{t}^{\mathrm{load}\_\mathrm{tran}}$$

where $\Delta P_{t}^{\mathrm{load}}$ denotes the load transfer power, and $P_{t}^{\mathrm{load}\_\mathrm{tran}}$ represents the load value after the load transfer.

(6) $$W_{t}^{\mathrm{load}}=C^{\mathrm{pen}}\cdot \left|\Delta P_{t}^{\mathrm{load}}\right|$$

where $W_{t}^{\mathrm{load}}$ denotes the load transfer compensation obtained by the user, and $C^{\mathrm{pen}}$ represents the unit price of the load transfer compensation.

### 2.3. Energy Storage Participating in Frequency Regulation Market

In the frequency regulation market, the compensation mechanism is based on capacity and service mileage to ensure that ESSs receive appropriate economic returns. The compensation for ESSs in the frequency regulation market can be calculated using the following equation [22]:

(7) $$W^{\mathrm{fm}}=K^{f}\cdot C^{f}\cdot P_{t}^{f}$$

where $W^{\mathrm{fm}}$ is the frequency regulation compensation provided by the frequency regulation market, and $K^{f}$ is the frequency regulation performance index, which typically includes three categories: frequency regulation time, frequency regulation mileage, and frequency regulation speed. Additionally, $C^{f}$ represents the ESS compensation unit price, and $P_{t}^{f}$ is the reported frequency regulation capacity. The frequency regulation compensation mechanism ensures that the ESS obtains a reasonable economic return, emphasizing its high responsiveness, adaptability to market price changes, and ability to dynamically adjust its capacity according to different needs [23].

Due to its exceptional power response characteristics, the ESS is often considered a high-quality resource for frequency modulation, and all resources intended for frequency modulation can be marketed accordingly. Therefore, while ensuring power balance within the park, a surplus ESS can be leveraged to participate in the frequency regulation market, thereby enhancing economic benefits and grid reliability [24].

During actual frequency modulation cycles, the modulation signal fluctuates between positive and negative values, averaging around zero. Sufficient capacity must be reserved in advance for the ESS to accurately respond to these signals in the frequency modulation market. Specifically, this involves the following:

(8) $$0\leq P_{i,t}^{\mathrm{ess}\_f}\leq P_{t}^{f}$$

where $P_{i,t}^{\mathrm{ess}\_f}$ is the frequency modulation signal that the ESS can respond to.

### 2.4. Economic Benefit Model of Park Area

**Figure 2:**
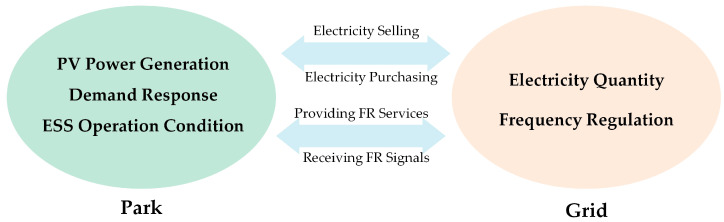
Diagram of park-to-grid interaction.

When considering the interaction between the PV storage park and the power grid for purchasing and selling electricity, as well as stabilizing park fluctuations, energy time shifting, and frequency regulation demands, the collaborative optimization strategy model must account for several factors. These include the energy and frequency regulation markets of park–grid interaction, the demand response of the park’s load side, and the operating conditions of the ESS. Figure 2 depicts this optimization strategy.

The power balance relationship within the park is established as shown in Equation (9):

(9) $$P_{t}^{\mathrm{load}}-\Delta P_{t}^{\mathrm{load}}=P_{t}^{\mathrm{pv}}+P_{t}^{\mathrm{bd}}-P_{t}^{\mathrm{bc}}+P_{t}^{g\_\mathrm{pv}}-P_{t}^{\mathrm{pv}\_g}$$

where $P_{t}^{g\_\mathrm{pv}}$ is the power sold by the park to the power grid, and $P_{t}^{\mathrm{pv}\_g}$ is the power purchased by the park from the power grid.

The park’s grid-interactive energy market comprises two main activities: selling electricity from the park to the grid and purchasing electricity. Specifically, electricity is purchased from the grid when the photovoltaic output falls short of meeting the load demand, and surplus electricity is sold to the grid when the photovoltaic output exceeds the park’s internal load and energy storage capacity [25]. This is illustrated below:

(10) $$\left\{ \begin{array}{c} P_{t}^{\mathrm{pv}\_g}=\max(0,P_{t}^{\mathrm{load}}+P_{t}^{\mathrm{bc}}-P_{t}^{\mathrm{pv}}-P_{t}^{\mathrm{bd}})\\ P_{t}^{g\_\mathrm{pv}}=\max(0,P_{t}^{\mathrm{pv}}+P_{t}^{\mathrm{bd}}-P_{t}^{\mathrm{load}}-P_{t}^{\mathrm{bc}}) \end{array}\right.$$

where the max function is used to ensure that the purchasing or selling power is zero or positive. Given that the ESS can only perform charging or discharging operations, the max function ensures that the purchased or sold power is always 0 or positive by selecting between 0 and the larger value from the internal calculation results of the expression.

The frequency modulation market primarily considers the compensation mechanism for frequency modulation and the operational strategies of the ESS. Introducing a demand response on the load side aims to increase the park operators’ revenue, reduce user expenses, and minimize the curtailed photovoltaic output. Through load transfer compensation, users are incentivized to shift electricity consumption from periods of low photovoltaic output to peak photovoltaic output periods. These market applications heavily rely on the operation of the ESS, with ESS efficiency being a critical concern for operators. Therefore, optimizing the utilization of the ESS to maximize profitability has emerged as a focal point of current research.

## 3. Dual-Layer Optimization Model for Integrated Operation of PV and ESS in Parks

To ensure the stability and quality of power supply, the ESS first smooths the fluctuations in photovoltaic output. The remaining ESS power is then optimized for collaborative operation between energy time shifting and frequency regulation to maximize the benefits of the photovoltaic park [26].

**Figure 3:**
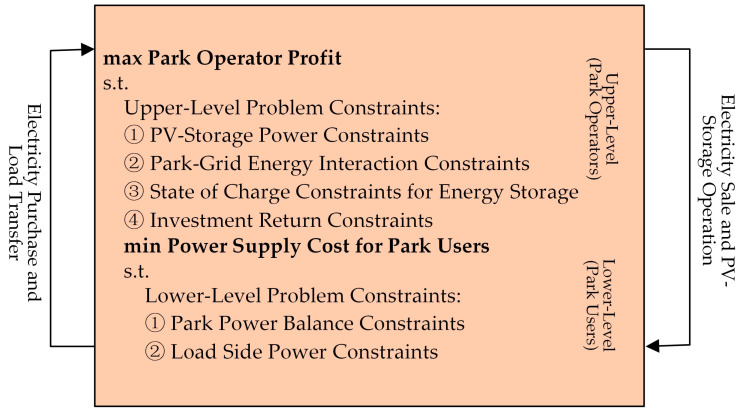
Dual-level optimization operation scheme.

The significance of dual-level planning lies in considering the interests of both upper-level operators and lower-level users. Therefore, a dual-level optimization model for the joint operation of a PV storage system in the park is established, as shown in Figure 3. To solve this max-min model, KKT conditions are used to transform the lower-level model into constraints of the upper-level model, and the solution provides optimized operation schemes for the park under different operation modes [27].

### 3.1. Optimization Model for Upper-Level Park Operators

#### 3.1.1. Objective Function

Considering the economic benefits for both park operators and users, the objective function for establishing an optimization strategy model for the joint operation of PV storage systems is as follows:

(11) $$\mathrm{Maximize}\;F=\sum_{t=1}^{24}(F_{t}^{\mathrm{se}}+F_{t}^{\mathrm{pv}\_g}+F_{t}^{\mathrm{fm}}-F_{t}^{\mathrm{ess}}-F_{t}^{g\_\mathrm{pv}})$$

where F is the total daily revenue of the park operator, $F_{t}^{\mathrm{se}}$ is the revenue from selling electricity to internal loads, $F_{t}^{\mathrm{pv}\_g}$ is the revenue from selling electricity to the grid, $F_{t}^{\mathrm{fm}}$ is the revenue from participating in the frequency regulation market, $F_{t}^{\mathrm{ess}}$ represents the ESS loss, and $F_{t}^{g\_\mathrm{pv}}$ is the expenditure on purchasing electricity from the grid.

(1) The revenue from load consumption within the park, including load consumption revenue and load transfer compensation, is calculated as follows:

(12) $$F_{t}^{\mathrm{pro}}=C^{\mathrm{user}}P_{t}^{\mathrm{load}}\Delta t-C^{\mathrm{pen}}\cdot \left|\Delta P_{t}^{\mathrm{load}}\right|$$

where $C^{\mathrm{user}}$ is the unit price of electricity for users in the park.

(2) The revenue from the energy interaction between the park and the grid, including park PV grid feed-in and park electricity purchases from the grid, is calculated as follows:

(13) $$\left\{ \begin{array}{l} F_{t}^{\mathrm{pv}\_g}=C^{\mathrm{pv}\_g}P_{t}^{\mathrm{pv}\_g}\Delta t\\ F_{t}^{g\_\mathrm{pv}}=C^{g\_\mathrm{pv}}P_{t}^{g\_\mathrm{pv}}\Delta t \end{array}\right.$$

where $C^{\mathrm{pv}\_g}$ is the on-grid electricity price of the park, and $C^{g\_\mathrm{pv}}$ is the electricity purchase price for the photovoltaic park from the power grid. The park’s feed-in tariff is divided into peak and off-peak periods during light-limiting and non-light-limiting periods, while the purchase tariff is divided into three periods, peak, off-peak, and flat, based on market demand characteristics.

(3) The economic lifespan loss of the ESS, including its participation in fluctuation mitigation, energy management, and the frequency regulation market, is calculated as follows:

(14) $$F_{t}^{\mathrm{ess}}=C^{b}(P_{t}^{e\_\mathrm{pv}}+P_{t}^{\mathrm{bc}}+P_{t}^{\mathrm{bd}}+P_{t}^{f})\Delta t$$

where $C^{b}$ is the unit price of ESS life loss.

#### 3.1.2. Constraint Condition

The constraints in the model primarily encompass power constraints between PVs and the ESS, energy interaction constraints between the grid and the park, load power constraints, state-of-charge constraints for the ESS, and investment return constraints [28].

(1) Power constraints for PVs and the ESS are as follows:

(15) $$\{\begin{array}{l} 0\leq P_{t}^{\mathrm{pv}}\leq P^{\mathrm{pv}\_\max}\\ 0\leq P_{t}^{e\_\mathrm{pv}}+P_{t}^{\mathrm{bc}}+P_{t}^{\mathrm{bd}}+P_{t}^{f}\leq P_{t}^{\mathrm{ess}\_\max} \end{array}$$

(2) Energy interaction constraints between the park and the grid are as follows:

(16) $$\left\{\begin{array}{l} 0\leq P_{t}^{\mathrm{pv}\_g}\leq P_{\max}^{\mathrm{pv}\_g}\\ 0\leq P_{t}^{g\_\mathrm{pv}}\leq P_{\max}^{g\_\mathrm{pv}}\\ P_{t}^{\mathrm{pv}\_g}\cdot P_{t}^{g\_\mathrm{pv}}=0 \end{array}\right.$$

(3) State-of-charge constraints for the ESS, operating within a specified range to extend the equipment’s lifespan, are as follows:

(17) $$\{\begin{array}{l} E_{t}^{\mathrm{ess}}=E_{t}^{\mathrm{ess}}+\left[\eta^{\mathrm{ch}}P_{t}^{\mathrm{bc}}-P_{t}^{\mathrm{bd}}/\eta^{\mathrm{dis}}\right]\Delta t\\ \begin{array}{l} 0.1E^{\mathrm{ess},\max}\leq E_{t}^{\mathrm{ess}}\leq 0.9E_{i}^{\mathrm{ess},\max}\\ E^{\mathrm{ess}}(24)=0.2E^{\mathrm{ess},\max} \end{array} \end{array}$$

where $E_{t}^{\mathrm{ess}}$ is the storage capacity of the ESS in time period *t*, $E^{\mathrm{ess},\max}$ is the maximum capacity of the ESS, $E^{\mathrm{ess}}(24)$ is the storage capacity at the end of the day, and $\eta^{\mathrm{ch}}$ and $\eta^{\mathrm{dis}}$ are the charging and discharging efficiencies of the ESS, both assumed to be 0.95.

(4) Return-on-investment constraints, ensuring profitability within the lifespan of PVs and the ESS, are as follows:

(18) $$\frac{C^{\mathrm{pv},p}P^{\mathrm{pv},\max}+C^{\mathrm{ess},p}P^{\mathrm{ess},\max}+C^{\mathrm{ess},e}E^{\mathrm{ess},\max}}{C^{\mathrm{pv}}E_{t}^{\mathrm{pv}}-\frac{C^{\mathrm{ess},p}P^{\mathrm{ess},\max}+C^{\mathrm{ess},e}E^{\mathrm{ess},\max}}{N_{2}\cdot n_{y}}}\leq N_{1}\cdot n_{y}$$

where $C^{\mathrm{pv},p}$ is the configuration cost of the PV power source, $C^{\mathrm{ess},p}$ is the power unit price of the ESS, $C^{\mathrm{ess},e}$ is the energy unit price of the ESS, $N_{1}$ is the PV investment payback period, $N_{2}$ is the operating life of the ESS, and $n_{y}$ is the number of days in a year.

### 3.2. Optimization Model for Lower-Level Park Users

#### 3.2.1. Objective Function

The lower-level model aims to minimize user costs, encompassing electricity expenses and load transfer compensation:

(19) $$\;\mathrm{Minimize}\;C=\sum_{t=1}^{24}(C^{\mathrm{user}}P_{t}^{\mathrm{load}}\Delta t-C^{\mathrm{pen}}\cdot \left|\Delta P_{t}^{\mathrm{load}}\right|)$$

#### 3.2.2. Constraint Condition

(1) Power balance constraints within the park are as follows:

(20) $$P_{t}^{\mathrm{load}}-\Delta P_{t}^{\mathrm{load}}=P_{t}^{\mathrm{pv}}+P_{t}^{\mathrm{bd}}-P_{t}^{\mathrm{bc}}+P_{t}^{g\_\mathrm{pv}}-P_{t}^{\mathrm{pv}\_g}:\lambda_{1,t}$$

(2) Power constraints on the load side, including load shifting requirements and limits, are as fololows:

(21) $$\left\{ \begin{array}{l} P_{t}^{\mathrm{load}}-L_{t}^{\mathrm{down}}\leq P_{t}^{\mathrm{load}\_\mathrm{tran}}\leq P_{t}^{\mathrm{load}}+L_{t}^{\mathrm{up}}:\mu_{1}^{\min},\mu_{1}^{\max}\\ 0\leq \max\left\{L_{t}^{\mathrm{down}},L_{t}^{\mathrm{up}}\right\}\leq \left(1-\delta \right)P_{t}^{\mathrm{load}}:\mu_{2}^{\min},\mu_{2}^{\max}\\ \sum_{t}P_{t}^{\mathrm{load}}=\sum_{t}P_{t}^{\mathrm{load}\_\mathrm{tran}}:\lambda_{2,t} \end{array}\right.$$

where $L_{t}^{\mathrm{down}}$ and $L_{t}^{\mathrm{up}}$ are the upper and lower limits of the transferable load, and δ is the minimum capacity reserve of the basic load during its transfer. $\lambda_{1,t}$ and $\lambda_{2,t}$ represent the Lagrange multipliers corresponding to the equality constraints, while $\mu_{1}^{\min}$, $\mu_{1}^{\max}$, $\mu_{2}^{\min}$, and $\mu_{2}^{\max}$ represent the Lagrange multipliers corresponding to the inequality constraints.

### 3.3. Model Solving

**Figure 4:**
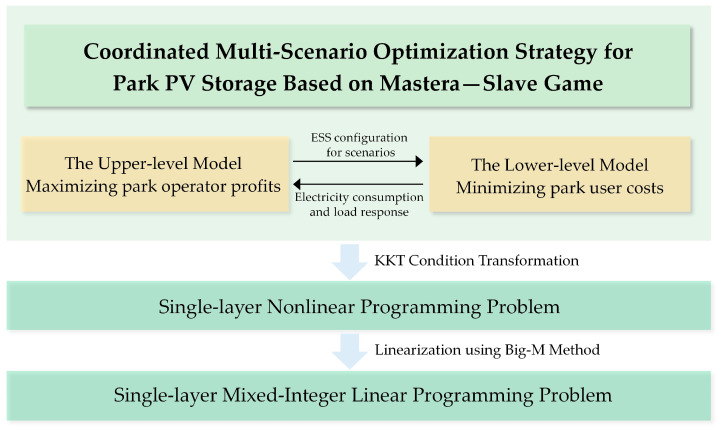
Dual-layer model solving process diagram.

The dual-layer model developed in this paper employs KKT conditions to embed the lower-level optimization model as constraints within the upper-level planning model. Additionally, the complementary relaxation conditions are linearized using the Big-M method to ensure computational feasibility [29,30]. Figure 4 illustrates the schematic diagram of the model solving process. The optimization model is implemented on the MATLAB platform utilizing the GUROBI 11.0 solver. The computation environment features an Intel(R) Core (TM) i7-12700H CPU (Lenovo Beijing, China) operating at 2.30 GHz with 32GB of memory.

This paper develops a Lagrangian function for the lower-level model, represented by Equation (22).

(22) $$\begin{array}{l} L=\sum_{t=1}^{24}(C^{\mathrm{user}}P_{t}^{\mathrm{load}}\Delta t-C^{\mathrm{pen}}\cdot \left|\Delta P_{t}^{\mathrm{load}}\right|)\\ +\sum_{t=1}^{24}\lambda_{1,t}(P_{t}^{\mathrm{pv}}+P_{t}^{\mathrm{bd}}-P_{t}^{\mathrm{bc}}+P_{t}^{g\_\mathrm{pv}}-P_{t}^{\mathrm{pv}\_g}-P_{t}^{\mathrm{load}}+\Delta P_{t}^{\mathrm{load}})\\ +\sum_{t=1}^{24}\lambda_{2,t}(P_{t}^{\mathrm{load}}-P_{t}^{\mathrm{load}\_\mathrm{tran}})\\ +\sum_{t=1}^{24}\mu_{1}^{\min}(P_{t}^{\mathrm{load}}-L_{t}^{\mathrm{down}}-P_{t}^{\mathrm{load}\_\mathrm{tran}})\\ +\sum_{t=1}^{24}\mu_{1}^{\max}(P_{t}^{\mathrm{load}\_\mathrm{tran}}+P_{t}^{\mathrm{load}}-L_{t}^{\mathrm{up}})\\ +\sum_{t=1}^{24}\mu_{2}^{\min}(-\max\{L_{t}^{\mathrm{down}},L_{t}^{\mathrm{up}}\})\\ +\sum_{t=1}^{24}\mu_{2}^{\max}(\max\{L_{t}^{\mathrm{down}},L_{t}^{\mathrm{up}}\}-(1-\delta )P_{t}^{\mathrm{load}}) \end{array}$$

Based on the constructed Lagrangian equation (22) and the KKT complementary relaxation condition for the lower-level model, the lower-level model can be transformed into additional constraints represented by Equations (23)—(24) for the upper-level model. The KKT direction and complementary conditions corresponding to the inequality constraints are as follows:

(23) $$\left\{ \begin{array}{l} 0\leq \mu_{1,t}^{\min}⊥P_{t}^{\mathrm{load}\_\mathrm{tran}}-P_{t}^{\mathrm{load}}+L_{t}^{\mathrm{down}}\geq 0\\ 0\leq \mu_{1,t}^{\max}⊥P_{t}^{\mathrm{load}}+L_{t}^{\mathrm{up}}-P_{t}^{\mathrm{load}\_\mathrm{tran}}\geq 0\\ 0\leq \mu_{2,t}^{\min}⊥\max\left\{L_{t}^{\mathrm{down}},L_{t}^{\mathrm{up}}\right\}\geq 0\\ 0\leq \mu_{2,t}^{\max}⊥\left(1-\delta \right)P_{t}^{\mathrm{load}}-\max\left\{L_{t}^{\mathrm{down}},L_{t}^{\mathrm{up}}\right\}\geq 0 \end{array}\right.$$

By introducing several 0–1 variables and employing the Big-M method, Equation (23) is transformed into a cutting plane constraint:

(24) $$\left\{\begin{array}{l} \langle \begin{array}{l} 0\leq \mu_{1,t}^{\min}\leq Mb_{1}\\ 0\leq P_{t}^{\mathrm{load}\_\mathrm{tran}}-P_{t}^{\mathrm{load}}+L_{t}^{\mathrm{down}}\leq M(1-b_{1}) \end{array}\\ \langle \begin{array}{l} 0\leq \mu_{1,t}^{\max}\leq Mb_{3}\\ 0\leq P_{t}^{\mathrm{load}}+L_{t}^{\mathrm{up}}-P_{t}^{\mathrm{load}\_\mathrm{tran}}\leq M(1-b_{3}) \end{array}\\ \langle \begin{array}{l} 0\leq \mu_{2,t}^{\min}\leq Mb_{5}\\ 0\leq \max\left\{L_{t}^{\mathrm{down}},L_{t}^{\mathrm{up}}\right\}\leq M(1-b_{5}) \end{array}\\ \langle \begin{array}{l} 0\leq \mu_{2,t}^{\max}\leq Mb_{5}\\ 0\leq \left(1-\delta \right)P_{t}^{\mathrm{load}}-\max\left\{L_{t}^{\mathrm{down}},L_{t}^{\mathrm{up}}\right\}\leq M(1-b_{5}) \end{array} \end{array}\right.$$

where *M* is a sufficiently large constant, and *b*_i_ is a binary variable. The slash bracket ⟨⟩ is used to indicate a set of included inequality constraints.

## 4. Case Study

### 4.1. Case Introduction

**Figure 5:**
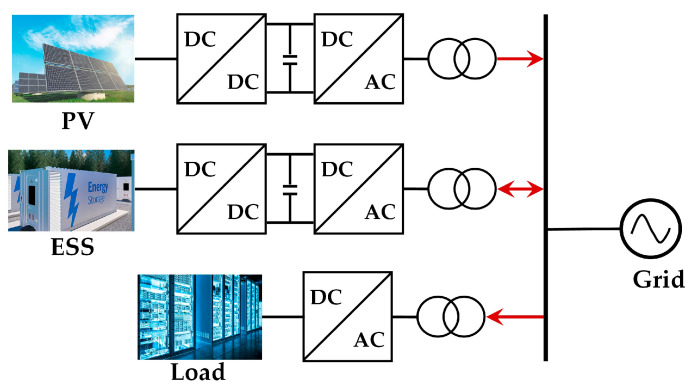
Simulation case model.

Based on data from a photovoltaic park situated in a mid-latitude city at 35 degrees north latitude, the photovoltaic output was simulated on typical cloudy summer days. The intermittent sunlight throughout the day caused the photovoltaic output to fluctuate with the weather conditions, accurately reflecting the characteristics of photovoltaic power generation. The simulation model is shown in Figure 5. The maximum rated power of the photovoltaic power station is 10 MW, and the ESS is configured at 2 MW/3 MWh. The energy storage is equipped with lithium–iron–phosphate batteries, with a power unit cost of 800 RMB/kW and an energy unit cost of 1800 RMB/kWh. The SOC is allowed to range from 10% to 90%, with a charging/discharging efficiency of 95% and an operational lifespan of 10 years. The configuration cost for the photovoltaic power source is 3000 RMB/kW and 2500 RMB/kW, with an investment payback period considered to be 5 years.

**Figure 6:**
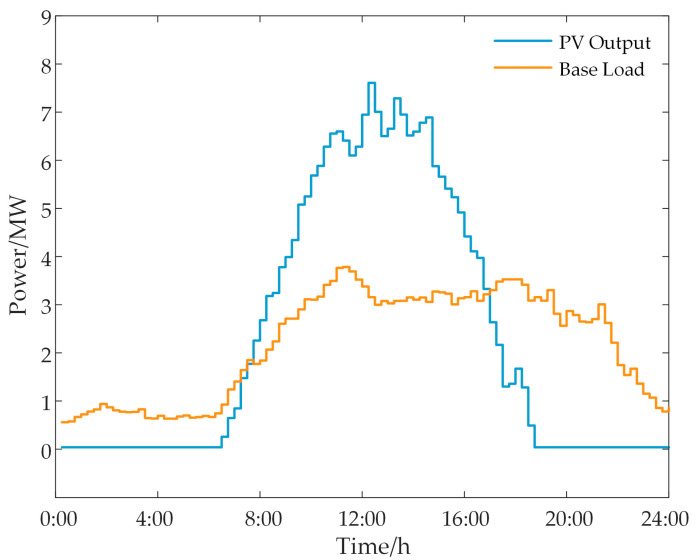
PV output and basic load.

The settings for the PV output limit and the basic load curve are illustrated in Figure 6. The load transfer fluctuation does not exceed 1 MW, and the degree of variation does not exceed 40%. During periods when light is limited, the power grid does not encourage photovoltaic grid connection, resulting in lower electricity prices. During non-light-limited periods, the power grid purchases PV energy at standard electricity prices. The electricity sale prices of the power grid are divided into three categories based on time nodes: peak, valley, and flat [31,32]. The settlement period for energy interaction and load compensation is 15 min. This approach dynamically adjusts and optimizes the energy flow and load distribution within the park, ensuring efficient operation and maximizing economic benefits.

The photovoltaic fluctuation characteristics are derived from the typical fluctuation patterns of PV arrays. The ESS employs full power compensation when addressing power fluctuation smoothing. The frequency modulation signal is based on the automatic generation control (AGC) signal from the power grid where the park is situated, and an equivalent AGC signal curve is generated accordingly. The frequency modulation signal has a power limit of 1.5 MW and operates daily from 6:00 to 23:00. The settlement period for frequency modulation compensation and energy storage life loss is set at 15 min, ensuring timely economic compensation and the long-term stable operation of the ESS. Table 1 details the various parameters of the model.

### 4.2. Case Analysis

#### 4.2.1. Optimization Results

**Figure 7:**
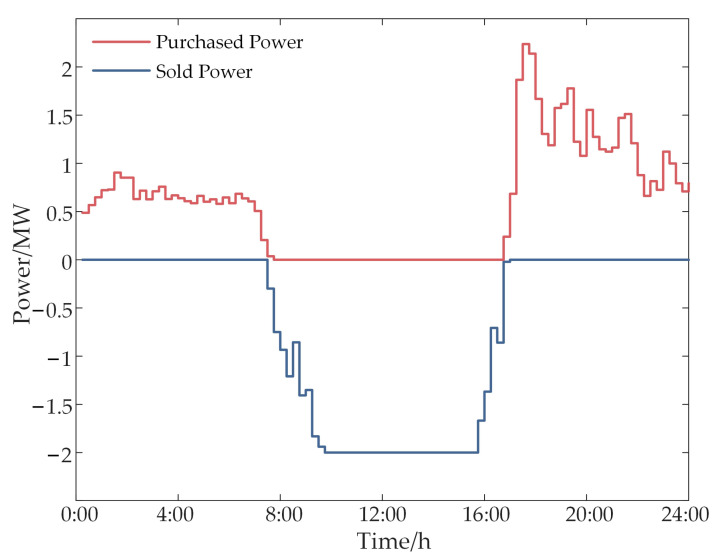
Park-to-grid energy exchange power.

Figure 7 illustrates the energy interaction power between the park and the power grid, indicating a maximum transmission limit of 2 MW from the park to the grid. During peak electricity pricing periods and when photovoltaic generation exceeds internal park demand, priority is given to meeting the park’s own electricity needs, with surplus electricity sold to the grid. At other times, discrepancies between the photovoltaic output and load demand necessitate the park to balance supply and demand through real-time electricity purchases.

**Figure 8:**
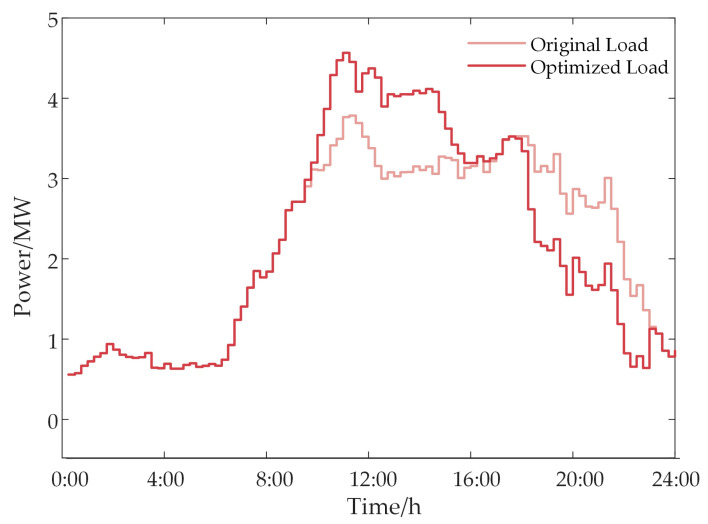
Load response changes.

Figure 8 depicts the load power curve before and after optimization, highlighting a notable shift in the load from the low valley period of PV power generation to its peak period. Through the implementation of a load response mechanism, users are effectively encouraged to increase electricity consumption during peak PV output periods. This strategy significantly enhances the park’s self-consumption rate of PV energy. At the same time, it reduces the reliance on electricity purchased from the grid.

**Figure 9:**
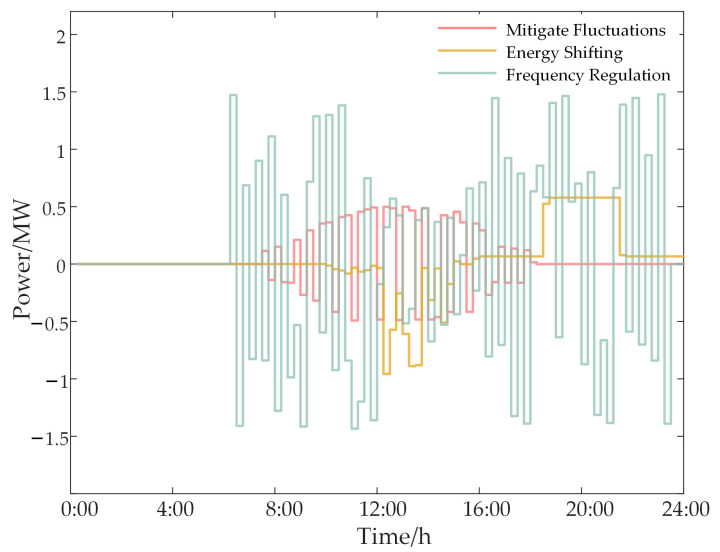
Power performance of ESS in three scenarios.

Figure 9 illustrates the operational performance of the ESS in three functions: PV fluctuation smoothing, energy time shifting, and frequency modulation markets. During noon periods, significant PV output fluctuations require the ESS to stabilize these variations to maintain system stability. Simultaneously, the system prioritizes energy time shifting over frequency modulation, as the economic benefits of time shifting exceed those of participating in frequency modulation, thereby optimizing ESS utilization.

**Figure 10:**
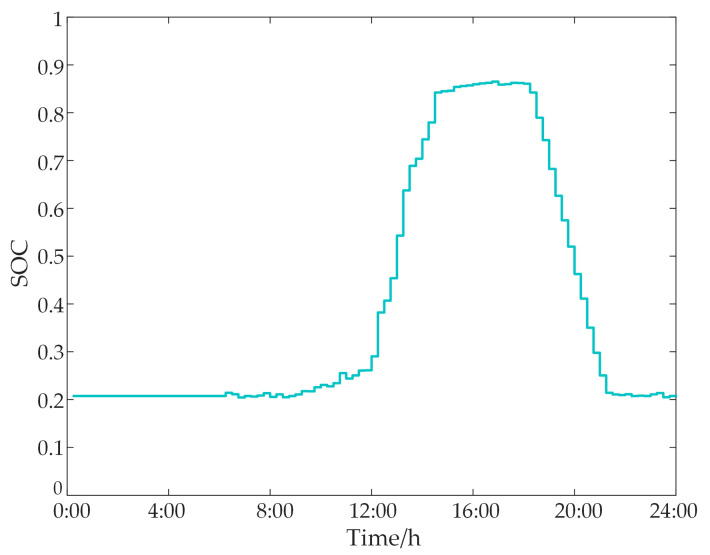
ESS SOC status.

Based on the SOC, initial SOC levels should be adjusted to enhance economic efficiency and reduce energy waste, thereby allowing for greater flexibility for future energy time shifting. Between 12:00 and 16:00, substantial PV energy is used to charge the ESS. Figure 10 indicates that park electricity sales reach their peak during this period. To maximize PV energy consumption and ensure adequate capacity for other applications, adjustments are necessary.

In conclusion, ESSs play a crucial role in coordinating PV fluctuation suppression, energy time shifting, and participation in the frequency modulation market. Effective configuration of ESS functionalities and statuses can significantly enhance PV energy utilization efficiency and overall economic benefits for the park.

#### 4.2.2. Objective Function of Upper-Level Model

To verify the effectiveness of the proposed operation strategy, three comparative scenarios are analyzed: the introduction of a load response mechanism within the park, the participation of PV storage in the frequency regulation market, and the various application scenarios discussed in this article. The optimization results are presented in Table 2 and Figure 7. As shown in Table 2, implementing energy time shifting within the park significantly reduces both electricity expenditures and photovoltaic power curtailment. Additionally, participating in the frequency regulation market markedly enhances the economic benefits for park operators.

Through the optimized operation combining both strategies, the total revenue for the park operators is calculated to be RMB 40,831.56, with the total expenditure for park users being RMB 34,534.67, and the amount of curtailed photovoltaic power being 0.813 MWh. Compared to solely implementing load response, the proposed optimization strategy enhances the economic benefit for the park operators by 25.8%, maintains user electricity expenditure at a comparable level, and keeps the curtailed photovoltaic power below 2%, significantly improving the economic efficiency of the park. In comparison to solely participating in the frequency regulation market, the proposed strategy increases the economic benefit for the park operators by 5.98%, reduces user electricity expenditure by 5.27%, and decreases the curtailment rate from 7.71% to 1.478%, significantly enhancing the utilization efficiency of photovoltaic power.

**Figure 11:**
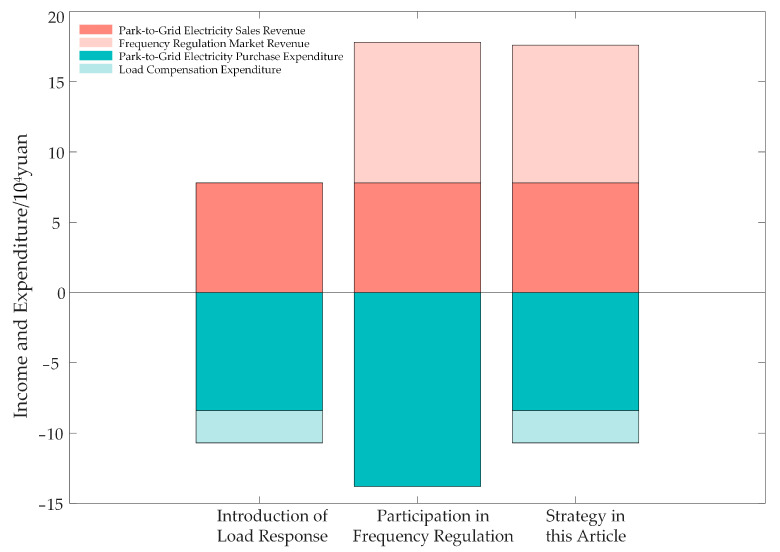
Revenue and expenditure under three operation strategies.

Figure 11 illustrates the revenue components for park operators under the three scenarios. Across various scenarios, the revenue from power sales to the grid remains relatively constant, indicating that the park’s power sales to the grid are near the maximum allowable limit for the entire period, leaving limited capacity for energy time shifting by the ESS. Without considering load-side response, the park power purchase expenditures increase due to the cancellation of load transfer compensation, leading to higher PV curtailment by the ESS. Simultaneously, to optimize economic returns, most of the ESS capacity is allocated to the frequency regulation market rather than to energy time shifting. Consequently, the optimization strategy achieves improved economic benefits primarily through increased revenue from the frequency regulation market, as well as reduced power purchase expenditures.

## 5. Conclusions

This paper proposes a multi-scenario collaborative optimization strategy for the operation of PV storage systems in parks based on a master–slave game model. It designs three types of energy storage application scenarios to comprehensively stabilize PV fluctuations, compensate for load transfers, and participate in the frequency regulation market, thereby optimizing the overall operational strategy of the park. The main findings from the simulation are summarized as follows:

(i) The proposed multi-scenario collaborative optimization strategy for PV storage systems in parks analyzes and derives the principles of PV fluctuation smoothing, load transfer compensation, and participation in the frequency regulation market, further summarizing the economic benefit model of the park.
(ii) The proposed master–slave game model for the joint operation of PV storage systems, based on the load response mechanism, reduces light abandonment. It increases frequency regulation market revenues and reduces electricity purchase expenditures, enhancing the economic benefits for park operators by 25.8% and effectively reducing user electricity costs by 5.27%, achieving better economic outcomes for both parties.

Future research will focus on analyzing the adjustment of strategy parameters under varying price conditions and exploring the optimization of ESS scheduling in different market price environments. Additionally, further investigations will be conducted on applying the proposed optimization strategy to the operation of electric vehicle charging stations to enhance overall economic benefits and grid stability.

## Figures and Tables

**Table 1:** Parameter values.

Parameter	Parameter Values
$C^{\mathrm{pen}}$/$C^{f}$/$C^{b}$	50/120/140
$C^{\mathrm{ess},p}$/$C^{\mathrm{ess},e}$	800/1800
$\eta^{\mathrm{ch}}$/$\eta^{\mathrm{dis}}$	0.95/0.95
*SOC*_max_/ *SOC*_min_	0.9/0.1
$N_{1}$/$N_{2}$	5/10

**Table 2:** Comparison of various operational strategies.

Scenario	Operating Revenue /Yuan	User Expenditure /Yuan	Abandoned PV Energy /MWh
introducing load response	32,460.75	34,893.26	0.386
participating in frequency regulation	38,526.42	36,456.24	4.237
multiple application scenarios in this paper	40,831.56	34,534.67	0.813

## Data Availability

The original contributions presented in this study are included in the article; further inquiries can be directed to the corresponding authors.
